# Commentary: Interlinking FinTech and eHealth: a qualitative study

**DOI:** 10.3389/fpubh.2026.1738604

**Published:** 2026-02-02

**Authors:** Larisha Janet Rodrigues, K. Dhivya Bharathi, Meghna Rajan, Haripriya Somasundaram, Anupriya Shanmugam, Ancy Antony, B. Sreya

**Affiliations:** 1 PSGR Krishnammal College for Women, Bharathiar University, Coimbatore, India; 2 PSG College of Arts and Science, Coimbatore, India; 3 KPR College of Arts Science and Research, Coimbatore, India; 4 Joy University, Tirunelveli, India

**Keywords:** benefits, digital payments, eHealth, FinTech, healthcare, interlink, Saudi Arabia

## 1 Introduction

The intersection of financial technology (FinTech) and electronic health (eHealth) has emerged as a transformative frontier in global public-health innovation. In “Interlinking FinTech and eHealth: A Qualitative Study,” Al-Anezi (1) explored this convergence through interviews with physicians, patients, and FinTech experts in Saudi Arabia, identifying both opportunities and barriers to digital-health transformation. The study revealed that integrating digital finance with healthcare systems improves efficiency, patient access, and financial inclusion, while regulatory gaps, data privacy, and the digital divide remain persistent challenges. While the original study is among the first qualitative inquiries to examine the FinTech and eHealth nexus in the Middle East, its implications extend far beyond the Saudi context. This commentary situates the study within the broader landscape of digital-health innovation, highlighting critical policy and equity concerns, proposing conceptual advances, and identifying new directions for empirical research.

## 2 Reframing the FinTech and eHealth interface

The original research reflects a global transition toward digital health economies, where financial infrastructures increasingly underpin healthcare accessibility. Digital payment systems, insurance automation, and telemedicine financing are no longer peripheral components but foundational pillars of sustainable health systems. However, understanding this intersection requires moving beyond efficiency-oriented narratives toward a systemic and data-governance perspective. Recent studies indicate that artificial intelligence (AI) is transforming health financing by optimizing governance, revenue generation, risk pooling, and strategic purchasing (2). Likewise, policy research on digital health technologies emphasizes that national frameworks, regulatory authorization, value assessment, pricing and reimbursement, and patient-access infrastructure are vital for achieving full-stack digital health integration (3). These insights affirm that FinTech and eHealth integration fosters inclusion and affordability, while underscoring the need for greater attention to data stewardship, cybersecurity, and ethical financial algorithms, dimensions only briefly explored in the original study.

## 3 Strengthening data governance and regulatory architecture

Participants in the original study identified regulatory complexity as the foremost barrier to FinTech and eHealth integration. This aligns with broader evidence showing that fragmented data regulations hinder interoperability across financial and healthcare domains. For example, recent digital-health governance research demonstrated that countries with strong digital infrastructures and coordinated governance frameworks are better equipped to ensure equitable access and system resilience (4). An actionable policy model is the European Health Data Space (EHDS), which proposes consent-based access, blockchain verification, and algorithmic accountability as cross-sector data-exchange mechanisms. Countries such as those in the Gulf Cooperation Council could adapt these approaches. Furthermore, adopting “RegTech-for-Health” frameworks, regulatory technologies that automate compliance monitoring and privacy enforcement in real time could enhance accountability and reduce compliance delays evident in slower-adoption contexts. However, the effectiveness of RegTech is contingent upon navigating regional regulatory diversity. While the Gulf Cooperation Council (GCC) utilizes centralized, vision-driven mandates to expedite digital transition, the European Union (EU) operates under a more rigid, decentralized privacy landscape (e.g., GDPR), which can necessitate different technical compliance architectures (5). In contrast, many South Asian models must contend with fragmented legal oversight, often requiring RegTech solutions to bridge significant gaps in formal digital-legal infrastructure.

## 4 From financial inclusion to digital health equity

A key strength of the original paper lies in its documentation of the benefits of financial inclusion, particularly among underinsured populations. However, inclusion alone does not necessarily translate into equity. As digital health financing expands, new forms of exclusion may emerge from algorithmic bias, limited digital literacy, and device dependency. A recent review of digital health start-ups and implementations found that structural factors such as service value, actor knowledge, and communication processes significantly influence equity outcomes (6). To advance health equity, FinTech-enabled health access should therefore be understood as part of a broader social justice agenda, one that emphasizes transparency, informed consent, device-agnostic design, and affordability safeguards. To move beyond a Eurocentric framing, the integration of FinTech and eHealth must account for the unique challenges of the Global South. In these regions, digital equity is frequently hampered by “infrastructure poverty,” including unreliable power grids and high costs for mobile data.Consequently, successful integration in the Global South often relies on “frugal innovation,” low-cost, high-impact technologies such as USSD-based mobile money wallets that operate on basic feature phones.By prioritizing these accessible gateways, developers can reach the “last mile” of the population who are traditionally excluded by the smartphone-centric models dominant in Western markets. Within Saudi Arabia's Vision 2030 framework, FinTech-driven health reforms should incorporate digital literacy initiatives targeting rural and older adults populations, thereby bridging the digital divide and reinforcing the original study's findings (7).

## 5 Technological synergies and the next phase of eHealth innovation

While the original article highlights future implications such as blockchain and wearables, this commentary emphasizes the need to conceptualize these as components of a “FinTech and eHealth ecosystem” rather than isolated tools. For example, a recent study described the convergence of FinTech and eHealth, illustrating how embedded systems and financial-risk engines are being used in healthcare settings to reduce costs and personalize user experiences (8). Furthermore, narrative reviews of digital-health technologies underscore the promise of AI, IoT, and cloud analytics while also highlighting the equity and regulatory risks if left unchecked (9). For successful integration, technical interoperability must be aligned with institutional interoperability, harmonizing policy, incentives, and workforce skills.

## 6 Methodological and conceptual considerations

The inductive qualitative design used in the original research was appropriate for exploring perceptions, yet future studies should advance toward cross-cultural and longitudinal analyses. FinTech and eHealth adoption is highly context-sensitive; comparative studies across Gulf countries, South Asia, and the European Union could illuminate how cultural attitudes toward digital trust shape adoption patterns. Conceptually, this commentary proposes extending the framework from “interlinkage” to a FinHealth Convergence Theory, which examines:

- Technological alignment (FinTech infrastructure embedded within eHealth platforms).
- Socio-economic alignment (financial inclusion aligned with health equity).
- Governance alignment (policy, ethics, and data security integrated across sectors).

Such a model can guide future empirical validation, providing the theoretical depth that the original study called for but did not elaborate (1).

## 7 Discussion and future directions

The integration of FinTech and eHealth represents more than digital modernization; it signals a restructuring of health-financing paradigms. The original study provides foundational insights into this transformation within Saudi Arabia, but several next-generation research and policy pathways are evident:

- Quantifying Societal Impact: Multi-country quantitative studies could measure outcomes such as reduced transaction delays, improved health access, and cost-savings, translating qualitative findings into scalable metrics (10).
- Embedding Ethics and AI Governance: Develop standardized frameworks for auditing algorithmic fairness in health-financing decision-making (2).
- Public–Private–Citizen Partnerships: Promote co-design of FinTech and eHealth solutions with end-users and communities to ensure trust and cultural alignment (7).
- Sustainability and Green Health Finance: Aligning FinTech-enabled healthcare with carbon-neutral digital infrastructure is emerging as a key focus in global health financing (4).

Ultimately, the future of FinTech and eHealth integration depends on reconciling innovation speed with governance depth. Policymakers must institutionalize digital trust through transparent data practices, cybersecurity investments, and inclusive financial models that place patient welfare above market efficiency.

## 8 Conclusion

The study under discussion effectively inaugurated scholarly discourse on FinTech-enabled eHealth in the Middle East. Building on its insights, this commentary advances a broader argument: the FinTech and eHealth nexus must evolve from a transactional model to a trust-centric, equity-driven digital-health ecosystem. By integrating financial inclusion with ethical AI, blockchain transparency, and adaptive regulation, healthcare financing can be reshaped into a more accessible, accountable, and resilient system. Future investigations should bridge qualitative insights with quantitative evaluation, enabling policymakers to design evidence-based digital-health finance strategies. By embedding inclusion, interoperability, and governance into the DNA of digital transformation, countries like Saudi Arabia can lead a global shift toward digitally equitable public health.
